# Early mobilization according to diagnosis in a Brazilian coronary ICU

**DOI:** 10.1186/cc14639

**Published:** 2015-03-16

**Authors:** GS Zavanelli, SA Padulla, MR Franco, RZ Pinto, LL Faccioli, DN Barbosa, DT Neves, CE Bosso

**Affiliations:** 1 Universidade Estadual Paulista - UNESP, Presidente Prudente, Brazil; 2 Instituto do Coração de Presidente Prudente, Brazil

## Introduction

Early mobilization has been advocated to improve muscle function and, consequently, the patient quality of life after discharge. Nevertheless, few studies have explored it in a coronary ICU (CICU). The aims of the present study were to describe the use of an early mobilization protocol in a CICU and to investigate whether different groups of diagnoses respond similarly to this protocol.

## Methods

This is a retrospective observational study conducted in a medium-sized hospital located in the city of Presidente Prudente, Brazil. The early mobilization protocol consists of five phases: 1 - passive exercises for the unconscious patient; 2 - active exercises associated with respiratory exercises (patient lying on the bed); 3 - phase 2 exercises with the patient sitting on the bed; 4 - phase 2 exercises with the patient sitting on a chair or in a standing position; 5 - phase 4 exercises plus walking. All hospital records from patients, between September 2013 and August 2014, were included in this study. Data extracted from hospital records were: age, gender, diagnosis (arrhythmia, coronariopathies, congestive heart failure and other pathologies), length of stay, number of discharge and number in each phase of the early mobilization protocol. Pearson chi-square test was used to compare the number of mobilizations (phase 4 and 5) per group of diagnoses. Odds ratios were calculated for those comparisons found to be statistically significant (*P *< 0.05).

## Results

A total of 697 hospitals records were analyzed. Patients had on average (SD) 67.8 (13.1) years and the majority of them were males (57%). Our results revealed that 65% of patients in the CICU received phase 4 and 43% of patients in the CICU received phase 5 of the early mobilization protocol. No differences in the proportion of patients receiving phase 4 or 5 were found among arrhythmia, coronariopathies and congestive heart failure groups. The only difference found was between congestive heart failure group and other cardiovascular pathologies (*P *< 0.001). The congestive heart failure group was mobilized 5.6 times (95% CI: 2.7 to 11.5) and 3.2 times (95% CI: 1.7 to 5.7) more than the other cardiovascular pathologies group in phase 4 and 5, respectively.

## Conclusion

A considerable proportion of patients was mobilized without any serious complications in the CICU. Our findings suggest that patients diagnosed with arrhythmia, coronariopathies and congestive heart failure can be equally mobilized in an ICU.

